# Long-term oncologic result of laparoscopic versus open gastrectomy for gastric cancer: a propensity score matching analysis

**DOI:** 10.1186/s12957-021-02217-2

**Published:** 2021-04-07

**Authors:** Si-Yuan Wu, Meng-Hsing Ho, Hao-Ming Chang, Kuo-Feng Hsu, Jyh-Cherng Yu, De-Chuan Chan

**Affiliations:** Division of General Surgery, Department of Surgery, Tri-Service General Hospital, National Defense Medical Center, 325 Chenggong Rd Sec 2, Nei-hu, 114 Taipei, Taiwan

**Keywords:** Laparoscopic surgery, Gastric cancer, Postoperative complications, Survival, Recurrence

## Abstract

**Background:**

Laparoscopic gastrectomy is an acceptable procedure for early-stage gastric cancer; however, most patients are diagnosed at an advanced stage and older age in Taiwan. The feasibility and safety of applying laparoscopic gastrectomy in daily practice remain unclear. This study aimed to examine the short- and long-term outcomes of laparoscopic gastrectomy versus open procedures.

**Methods:**

From 2007 to 2015, 192 patients who underwent open gastrectomy and 189 patients who underwent laparoscopic gastrectomy for gastric cancer at a single center were included. Propensity score matching analysis was used to adjust selection biases associated with age, preoperative hemoglobin, the extent of resection, tumor size, and stage of the disease. The demographics, perioperative parameters, short-term postoperative results, and 5-year survival data were analyzed.

**Results:**

Open gastrectomy was more frequently performed in the elderly, larger tumor size, advanced stage of the disease, and disease requiring total gastrectomy or combined organ resection. After propensity score matching, 108 patients with laparoscopic gastrectomy were compared to 108 patients with open gastrectomy. The morbidity rates were not different in both groups (25.9%), while hospital stay was shorter in the laparoscopic group (16.0 vs. 18.8 days, *p* = 0.04). The 5-year overall survival and disease-free survival were superior in the laparoscopic group (*p* = 0.03 and *p* = 0.01, respectively); however, the survival differences were not significant in the subgroup analysis by stage. Laparoscopic gastrectomy had fewer recurrences than open gastrectomy. The pattern of recurrence was not different between the groups.

**Conclusions:**

Laparoscopic gastrectomy can be safely applied in both early and locally advanced gastric cancer without compromising oncologic outcomes.

**Trial registration:**

Retrospective registration.

## Background

Gastric cancer (GC) is the fourth leading cause of cancer-mediated death worldwide and is more prevalent in Asian countries [1]. Surgery with radical gastrectomy and lymph node dissection is the single most effective treatment for localized, non-metastatic disease. After 1990, the application of laparoscopy in cancer surgery was emerging. Since the initial application in colectomy, laparoscopic surgery was frequently associated with reduced surgical stress and faster recovery than the traditional open procedure [2]. However, safety and oncologic integrity concerns would continue until its performance to be evaluated in general practice, especially the adequacy of lymphadenectomy. In the last decade, several randomized controlled trials [3–7], including phase 3 trials [8–11] have indicated the feasibility of laparoscopic gastrectomy (LG) for treating early-stage GC. Compared to conventional open gastrectomy (OG), LG is associated with reduced blood loss, faster recovery, and a similar number of retried lymph nodes but a longer operative time in these studies. Moreover, available data on long-term survival did not show statistical differences between laparoscopic and open surgery [12, 13]. In recent years, an increasing number of reports from specialized centers in Asian countries, including multicenter randomized trials [14, 15], have demonstrated that LG is technically safe for locally advanced disease without compromising the quality of lymph node dissections. Long-term survival data from these large-scale studies are ongoing, although recent data have shown promising results [16–18].

Since patients with early GC are frequently asymptomatic, except for some East Asian countries where national screening is widely performed, most patients are diagnosed at advanced stages [19]. In Taiwan, most GC patients are diagnosed at an advanced stage. Among patients who can be operated on, 68.1% had stage > II, and 48.9% were of age ≥ 70 years [20]. However, most studies regarding the safety of LG often include younger patients with few comorbidities. Older patients may benefit from the smaller incision and shorter hospital stay following laparoscopic surgery. Still, the advantages could also be offset by specific physiologic stress from pneumoperitoneum and longer operative time [21]. The feasibility and safety of LG in daily practice, which involves mostly treating elderly patients and advanced stage diseases, remains unclear.

At our institute, we expanded our indication and adopted laparoscopic surgery as the primary procedure for advanced GC as our experience developed. In recent years, either advanced age or the cancer stage did not necessarily preclude GC patients from LG. This practice had made our laparoscopic cohort closer to daily practice. However, a direct comparison between laparoscopic and open surgery would be unjustified if selection biases were not adjusted. In this study, we aimed to compare the outcomes between LG and OG for GC using propensity score matching analysis.

## Methods

We conducted a single-center, retrospective cohort study that included GC patients initially treated by radical gastrectomy at the Tri-Service General Hospital from January 2007 to December 2015. All patients were included except those with palliative procedures and open conversions. Clinical data were gathered from chart reviews after approval by the institutional review board of the Tri-Service General Hospital (TSGHIRB No. 1-106-05-098).

Patients were diagnosed with GC via endoscopic biopsy and went through subsequent staging workup, including contrast computed tomography (CT) scans, to evaluate the extent of tumor involvement, as well as nodal status, and to exclude distant metastasis. Endoscopic ultrasound was only performed to assess the feasibility of endoscopic mucosal resection or endoscopic submucosal dissection in clinically advanced GC. Gastrectomy with lymph node dissection was the standard treatment for the locoregional disease. Patients eligible for surgery were assessed for comorbid conditions, nutritional state, and functional status. In patients with high perioperative risk, such as age over 80, American Society of Anesthesiologists (ASA) classification ≥ 3, or Eastern Cooperative Oncology Group (ECOG) performance status ≥ 2, the informed consent process involved anesthesiologists and cardiologists. Alternative palliative treatment options and associated prognostic data were also provided.

During the informed consent process before the operation, the surgeons informed the candidates regarding the pros and cons of LG as well as the costs, after which the candidates decided between laparoscopic or open surgery. A diagnostic laparoscopy always preceded surgery to evaluate resectability. For localized resectable disease, the surgeon proceeded to perform OG or LG based on the patient’s decision. The absolute contraindications to laparoscopic surgery were (1) tumors with direct invasion of the duodenum, pancreatic head, or esophagus; (2) bulky lymph nodes; (3) unstable hemodynamics during surgery.

### Treatment

All laparoscopic and open surgeries were performed by a single surgeon (DC Chan). The decision between distal or total gastrectomy was dependent on tumor location—a minimum of 5 cm of proximal safe margin was required. A D2 lymphadenectomy, according to Japanese guidelines [22], was the standard extent of lymph node dissection, while the extent could be D1 or D1+ in aged, comorbid patients, or in those with clinically early GC. In total gastrectomy for proximal GC, splenectomy was not routinely included in D2 lymphadenectomy, except if the tumor was adjacent to the splenic hilum or involved No. 10 or 11 lymph nodes. Combined organ resection was performed when the tumor grossly invaded the adjacent organs. The laparoscopic procedure started with the patient in the supine position with legs spread apart. Four trocar ports were created and arranged in a curved pattern: one 12-mm camera port at the umbilicus; one 5-mm and one 12-mm operator port at each side; and another 5-mm assistant port at the left subcostal region. Pressure for pneumoperitoneum was 12 mmHg. The dissection began at the greater curvature side, from the left to right gastroepiploic region, including total omentectomy and regional lymphadenectomy (No. 4sb, 4d, 6). The duodenum was transected by a linear stapler. Lymphadenectomy was then performed along the hepatoduodenal ligament (No. 12a), suprapancreatic region (No. 8a, 9, 11p), and lesser curvature side (1, 3, 5, 7). For distal gastrectomy, gastrojejunostomy was anastomosed using linear staplers in Roux-en-Y fashion after dividing the stomach. For total gastrectomy, esophagojejunostomy was anastomosed using a circular stapler in Roux-en-Y fashion after dividing the esophagus. Either procedure was anastomosed intracorporeally without additional incision. The specimen was retrieved from the extended umbilical incision with a wound protector. The sequences of the procedure were basically the same for both LG and OG.

Postoperative care was the same as for both approaches. Abdominal drains and Foley’s catheter were routinely placed, but a nasogastric tube was usually not required. Patients aged over 80 years, with severe comorbidities, or who underwent lengthy procedures for extensive resection generally had postoperative intensive care. Patients were administered a liquid diet after the first flatus. Hospital discharge required that patients could tolerate an adequate diet and had sufficient control of medical diseases.

The patient surveillance and subsequent adjuvant chemotherapy after surgery were conducted by the same surgeon. Except for patients with early GC or contraindications, all patients underwent adjuvant chemotherapy. The regimen was generally capecitabine plus oxaliplatin, with or without paclitaxel. A follow-up was scheduled at least every 3 months in the first 3 years after completion of treatment. Laboratory studies, including tumor markers, were checked at each follow-up visit, and abdominal ultrasound was performed every 6 months. CT scans were used to confirm recurrence if there was any clinical suspicion during surveillance.

### Outcomes evaluation

The short-term outcome included operative time, hospital stay, and early complications. The operative time was correlated with the chronological order of operation by scatter plot with linear regression to examine any experience curve effect. Early complications referred to adverse events within 30 days of surgery and were graded by extended Clavien-Dindo classification [23]. Complications confined to the abdomen were categorized into “local,” which were related to the gastric resection, otherwise “systemic.” Delayed gastric emptying was defined as gastric stasis that required nasogastric tube drainage at the tenth postoperative day. Dumping syndrome was defined as repeated gastrointestinal discomforts, perspiration, or decreased consciousness level after meals. Ileus was defined as the inability to tolerate oral intake on the seventh postoperative day due to bowel motility impairment. The intraabdominal abscess was referred to localized pus collection within the peritoneal cavity, and it was differentiated from the chylous ascites by the presence of triglyceride above 200 mg/dL. Postoperative bleeding included intraluminal or extraluminal bleeding that required blood transfusion or intervention. Anastomotic leakage was referred to as leakage of intraluminal contents to the peritoneal cavity detected by abdominal drains, cross-sectional image studies using gastrointestinal contrast, or reoperative findings. The long-term outcomes were 5-year overall survival (OS) and disease-free survival (DFS), which were calculated from the date of surgery. The last follow-up date was October 31, 2020.

### Statistical analysis

All analyses were performed using the R 3.5.2 software (R Foundation for Statistical Computing, Vienna, Austria). Differences were considered significant at two-sided *p* values < 0.05. Descriptive statistics were expressed as mean ± standard deviation for continuous variables and frequency (percentage) for categorical variables. Univariate analysis was performed using the Mann-Whitney *U* test for continuous variables and Chi-square test or Fisher’s exact test for categorical variables.

Propensity score matching was applied to minimize the imbalances associated with selection biases. In the model, propensity scores were developed, accounting for all factors significantly associated with either undergoing LG or OG. Accordingly, individual propensity scores were calculated through logistic regression modeling based on the following covariates: age, preoperative hemoglobin, total gastrectomy, combined organ resection, tumor size, closest surgical margin, number of harvested lymph nodes, and stage. Patients undergoing LG and OG were then paired 1:1 on these propensity scores using nearest matching with caliper size = 0.1. Balances between the groups were examined by graphical approaches, jitter plot, and back-to-back histogram.

Following propensity score matching, the short-term outcome between LG and OG was compared. The long-term survival between the two groups was estimated by the Kaplan-Meier method and compared by log-rank test.

## Results

### Determinants that affect the selection of procedures

**Fig. 1:**
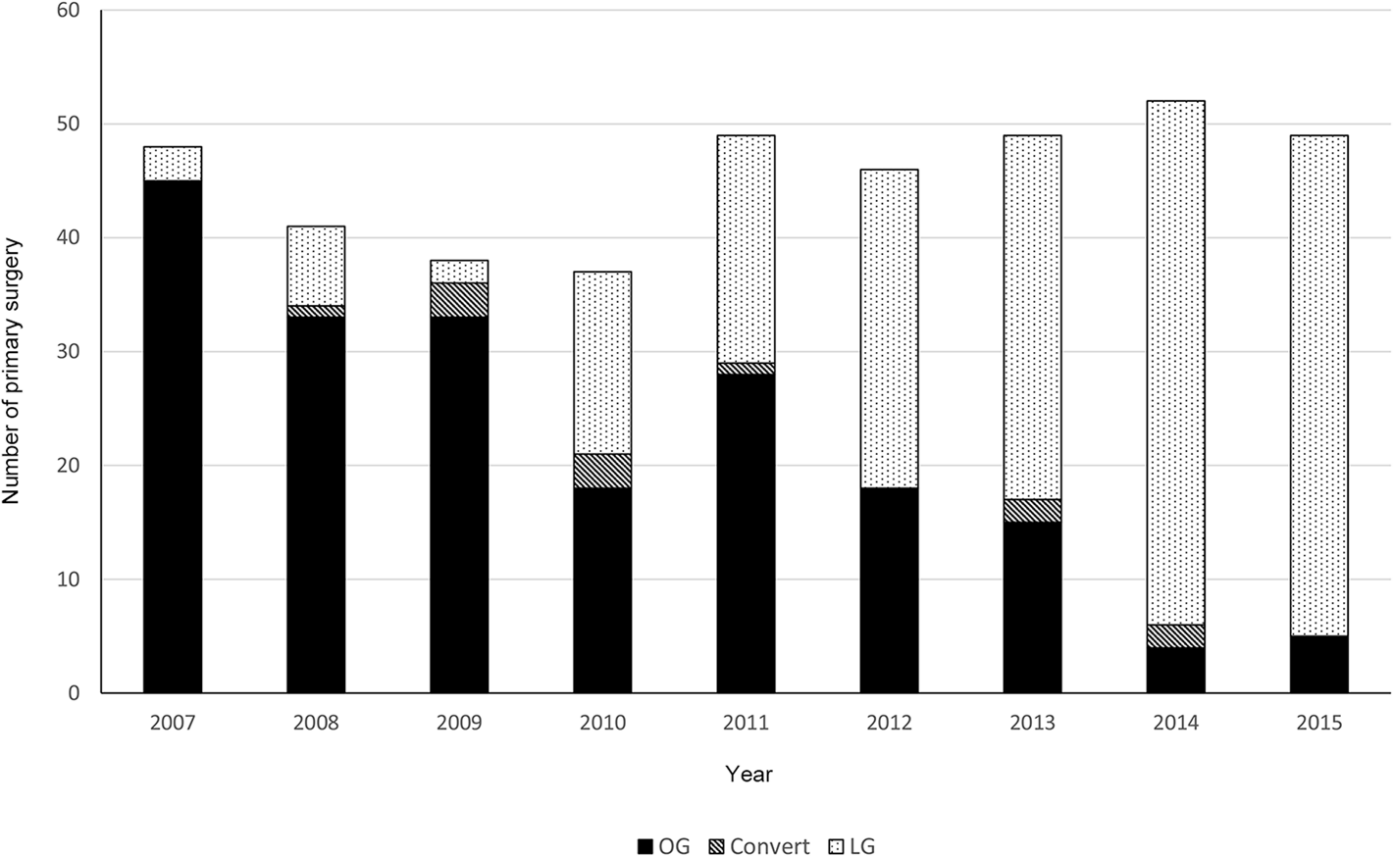
The year-wise change in surgical practice. Laparoscopic gastrectomy became the primary treatment for gastric cancer over the years. OG, open gastrectomy; LG, laparoscopic gastrectomy

The year-wise changes in surgical practice and distribution of LG are shown in Fig. 1. After excluding 16 palliative surgeries and 12 conversions, a total of 381 consecutive patients were included. This included 192 patients with OG and 189 with LG (Table 1). The rate of advanced GC treated by laparoscopic approach increased by years, which had approached 90% after 2014.

**Table 1:** Comparison of perioperative variables between cohorts underwent OG or LG

	Before propensity score matching			After propensity score matching		
	OG (*n*=192)	LG (*n*=189)	*p* value	OG (*n*=108)	LG (*n*=108)	*p* value
Age	67.9±1.0	64.6±1.1	0.01	67.6±1.3	67.8±1.5	1.00
≧ 70	97 (50.5%)	76 (40.2%)	0.04	53 (49.1%)	56 (51.9%)	0.68
Sex: male	135 (70.3%)	120 (63.5%)	0.16	74 (68.5%)	69 (63.9%)	0.47
Body mass index	24.2±0.3	24.0±0.2	0.48	24.4±0.3	23.5±0.3	0.03
Hemoglobin	11.0±0.2	12.4±0.1	<0.01	11.7±0.2	11.8±0.2	0.96
ASA class			0.66			0.30
1	61 (31.8%)	52 (27.5%)		36 (33.3%)	28 (25.9%)	
2	97 (50.5%)	101 (53.4%)		53 (49.1%)	53 (49.1%)	
3	34 (17.7%)	36 (19.0%)		19 (17.6%)	27 (25.0%)	
Charlson comorbidity index	4.7±0.1	4.6±0.1	0.58	4.6±0.2	4.9±0.2	0.17
Prior abdominal surgery	26 (13.5%)	35 (18.5%)	0.19	15 (13.9%)	18 (16.7%)	0.57
Extent of gastrectomy			<0.01			1.00
Distal gastrectomy	113 (58.9%)	142 (75.1%)		77 (71.3%)	77 (71.3%)	
Total gastrectomy	79 (41.1%)	47 (24.9%)		31 (28.7%)	31 (28.7%)	
Extent of lymphadenectomy			0.25			0.58
Less than D2	37 (19.3%)	28 (14.8%)		19 (17.6%)	16 (14.8%)	
D2	155 (80.7%)	161 (85.2%)		89 (82.4%)	92 (85.2%)	
Combined organ resection	41 (21.4%)	8 (4.2%)	<0.01	10 (9.3%)	8 (7.4%)	0.62
Tumor size (mm)	52.1±2.0	41.9±1.7	<0.01	49.1±2.8	48.0±2.4	0.85
Retrieved lymph nodes	25.4±1.1	30.2±1.1	<0.01	28.7±1.4	28.4±1.4	0.99
Metastatic lymph nodes	5.1±0.5	2.9±0.4	<0.01	3.6±0.5	3.6±0.5	0.96
Closest resection margin	28.5±1.2	34.4±1.1	<0.01	30.6±1.6	30.5±1.4	0.72
Pathologic T stage			<0.01			0.17
1	28 (14.6%)	69 (36.5%)		23 (21.3%)	29 (26.9%)	
2	27 (14.1%)	37 (19.6%)		22 (20.4%)	18 (16.7%)	
3	44 (22.9%)	43 (22.8%)		23 (21.3%)	33 (30.6%)	
4	93 (48.4%)	40 (21.2%)		40 (37.0%)	28 (25.9%)	
Pathologic N stage			<0.01			0.56
0	66 (34.4%)	108 (57.1%)		50 (46.3%)	49 (45.4%)	
1	31 (16.1%)	28 (14.8%)		15 (13.9%)	19 (17.6%)	
2	40 (20.8%)	23 (12.2%)		24 (22.2%)	17 (15.7%)	
3	55 (28.6%)	30 (15.9%)		19 (17.6%)	BBG	
Pathologic stage			<0.01			0.68
I	40 (20.8%)	94 (49.7%)		35 (32.4%)	41 (38.0%)	
II	44 (22.9%)	38 (20.1%)		25 (23.1%)	22 (20.4%)	
III	108 (56.3%)	57 (30.2%)		48 (44.4%)	45 (41.7%)	
Lymphovascular invasion	102 (53.1%)	66 (34.9%)	<0.01	51 (47.2%)	46 (42.6%)	0.49
Extracapsular extension	72 (37.5%)	38 (20.1%)	<0.01	28 (25.9%)	28 (25.9%)	1.00
Poor differentiated	151 (78.6%)	134 (70.9%)	0.08	77 (71.3%)	75 (69.4%)	0.77
Adjuvant chemotherapy	74 (38.5%)	64 (33.9%)	0.34	36 (33.3%)	40 (37.0%)	0.57

*OG* open gastrectomy, *LG* laparoscopic gastrectomy, *ASA Class* American Society of Anesthesiologists Classification

Patients who underwent LG were younger (64.6 vs. 67.9, *p* = 0.01) and had a higher preoperative hemoglobin (12.4 vs. 11.0 mg/dL, *p* < 0.01), but were not different from those who underwent open surgery in terms of preoperative comorbidities. Open surgeries were more frequently applied in larger tumor size (52.1 vs. 41.9 mm, *p* < 0.01), diseases that required total gastrectomy (41.1 vs. 24.9%, *p* < 0.01), and more locally advanced diseases (T4: 48.4 vs. 21.2%, *p* < 0.01; above N1: 49.4 vs. 28.1%, *p* < 0.01; stage 3: 56.3 vs. 30.2%, *p* < 0.01).

### Characteristics of propensity score-matched cohorts

Patients were matched 1:1 based on determinants affecting the selection of procedures as described above. The propensity score-matched cohort from the analysis included 216 patients: 108 in the OG group versus 108 in the LG group (Table 1). Covariates imbalance observed in the previous analysis were alleviated after matching, except for the lower body mass index (BMI) in the LG group (23.5 vs. 24.4, *p* = 0.03). The matched cohorts were not statistically different in terms of age, sex, preoperative comorbidity status, the extent of gastrectomy or lymph node dissection, stage, or rate of postoperative adjuvant chemotherapy.

### Comparison of short-term outcome in the propensity score-matched cohorts

**Fig. 2:**
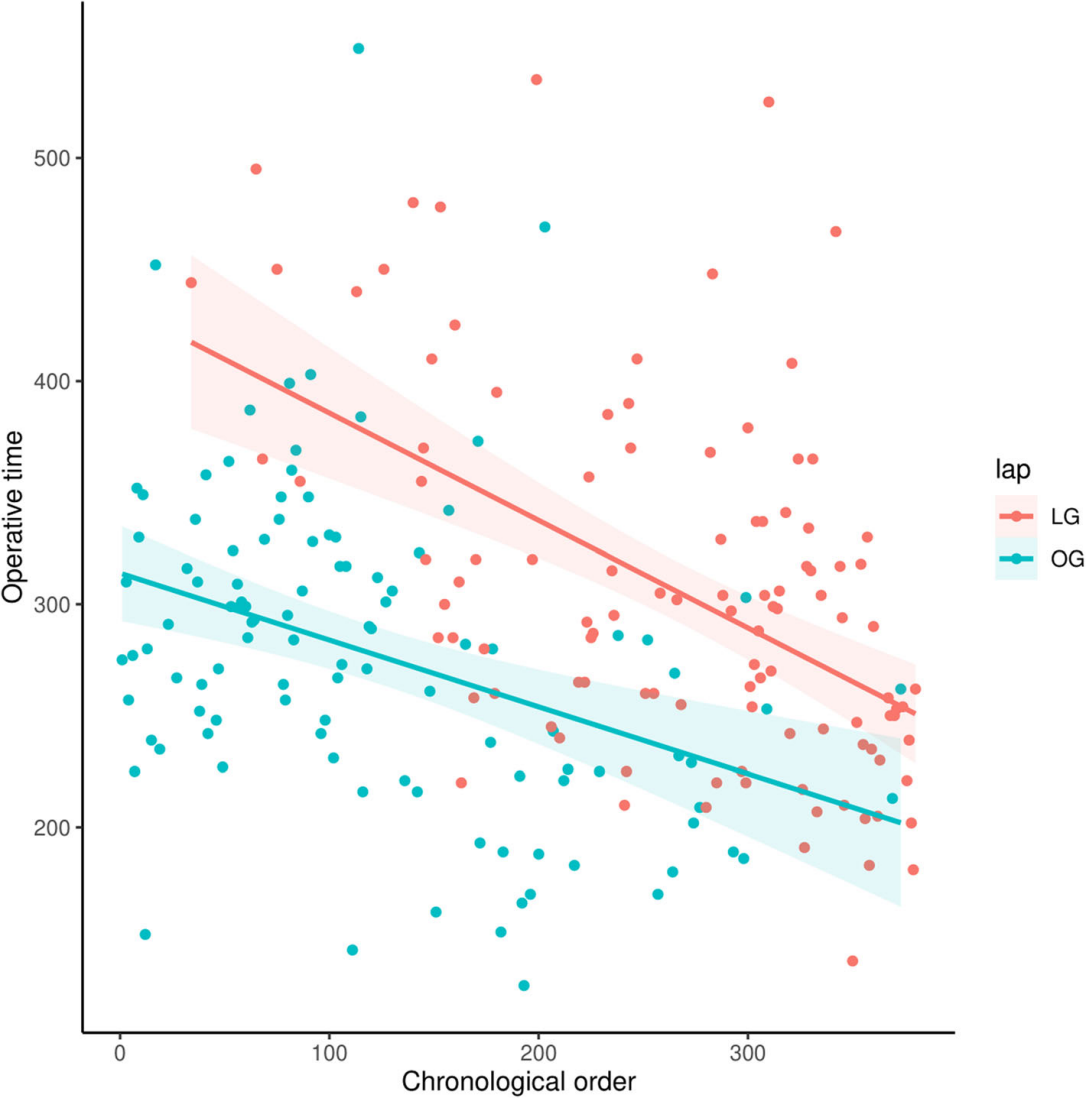
The operative time versus chronological order of operation in scatter plot. The operative time was decreasing for both OG (green) and LG (red). Solid line: regression line by linear regression for each group of data, shadow: 95% confidence interval

The short-term outcome is shown in Table 2. The LG group had a longer operative time (305.3 vs. 277.4 min, *p* = 0.03), but shorter hospital stays (16.0 vs. 18.8 days, *p* = 0.041). While the operative time was correlated with the chronological order of operation, both groups showed decreasing trends, with a similar slope of regression lines (Fig. 2). In addition, both groups had the same rate of early complication (25.9%) with a similar rate of severe complications (Clavien-Dindo classification ≥ 3: 7.4% for OG vs. 6.5% for LG, *p* = 0.79). By individual event, OG tended to have more dumping syndrome complications, while LG tended to have more pulmonary complications; however, none of the differences had statistical significance.

**Table 2:** Comparison of short-term outcome in the propensity score-matched cohort

	Overall			Distal gastrectomy			Total gastrectomy		
	OG (*n*=108)	LG (*n*=108)	*p*	OG (*n*=77)	LG (*n*=77)	*p*	OG (*n*=31)	LG (*n*=31)	*p*
Operative time	277.4±6.8	305.3±7.8	0.03	267.2±7.3	294.1±9.1	0.11	302.6±14.7	333.1±13.9	0.15
Hospital stay	18.8±1.7	16.0±0.9	0.04	17.9±2.2	15.2±1.1	0.02	20.8±2.7	17.9±1.6	0.68
Overall complications	28 (25.9%)	28 (25.9%)	1.00	21 (27.3%)	22 (28.6%)	0.86	7 (22.6%)	6 (19.3%)	0.76
Clavien Dindo class ≥3	8 (7.4%)	7 (6.5%)	0.79	6 (7.8%)	4 (5.2%)	0.51	2 (6.5%)	3 (9.7%)	0.64
Local complications									
Delayed gastric emptying	3 (2.8%)	4 (3.7%)	1.00	3 (3.9%)	4 (5.2%)	0.70	0	0	
Dumping syndrome	5 (4.6%)	0	0.06	4 (5.2%)	0	0.12	1 (3.2%)	0	1.00
Ileus	4 (3.7%)	4 (3.7%)	1.00	3 (3.9%)	3 (3.9%)	1.00	1 (3.2%)	1 (3.2%)	1.00
Chylous ascites	1 (0.9%)	2 (1.9%)	1.00	1 (1.3%)	2 (2.6%)	1.00	0	0	
Bleeding	0	0		0	0		0	0	
Intraabdominal abscess	6 (5.6%)	4 (3.7%)	0.75	5 (6.5%)	4 (5.2%)	0.73	1 (3.2%)	0	1.00
Anastomotic leakage	1 (0.9%)	0	1.00	0	0		1 (3.2%)	0	1.00
Wound infection	2 (1.9%)	2 (1.9%)	1.00	1 (1.3%)	1 (1.3%)	1.00	1 (3.2%)	1 (3.2%)	1.00
Systemic complications									
Pulmonary complications	5 (4.6%)	11 (10.2%)	0.193	4 (5.2%)	5 (6.5%)	0.73	1 (3.2%)	6 (19.4%)	0.10
Cardiovascular events	u1 (0.9%)	2 (1.9%)	1.00	1 (1.3%)	2 (2.6%)	1.00	0	0	
CRBSI	3 (2.8%)	2 (1.9%)	1.00	2 (2.6%)	2 (2.6%)	1.00	1 (3.2%)	0	1.00

*CRBSI* catheter-related bloodstream infection

In the subgroup analysis for distal and total gastrectomy, the two groups were not different statistically in terms of complication rates, neither overall nor by events (Table 2). In the subgroup analysis for the elderly (patient age ≥70), the overall complication rates were similar (30.2% for OG vs. 28.6% for LG, *p* = 0.85).

### Comparison of the long-term outcome in the propensity score-matched cohorts

**Fig. 3:**
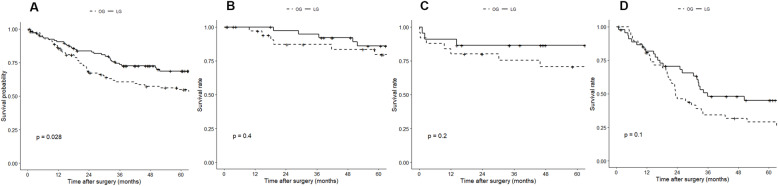
The 5-year overall survival after laparoscopic versus open gastrectomy estimated by the Kaplan-Meier method. **a**, all stages; **b**, pathologic stage 1; **c**, pathologic stage 2; **d**, pathologic stage 3

**Fig. 4:**
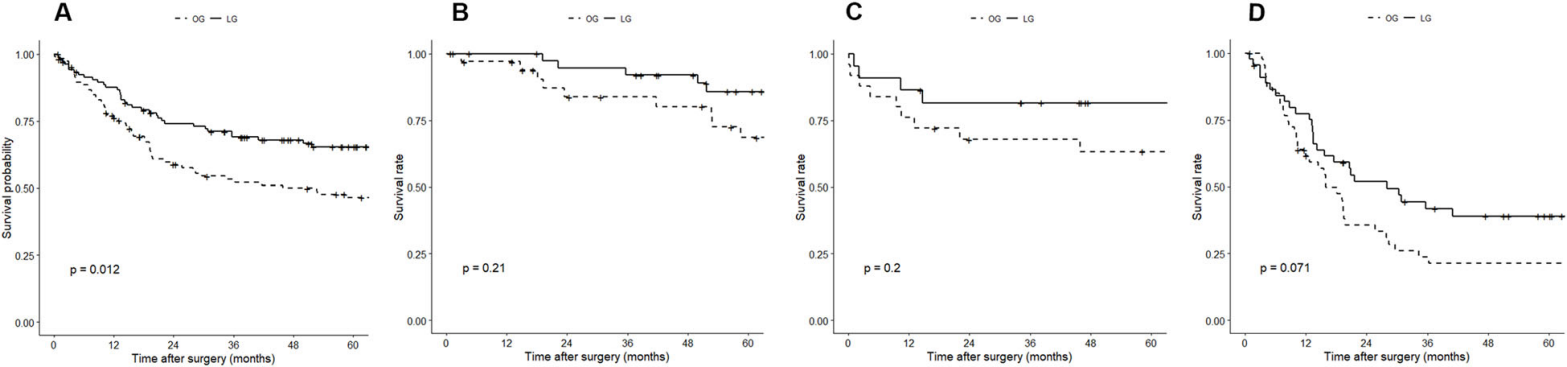
The 5-year disease-free survival after laparoscopic versus open gastrectomy estimated by the Kaplan-Meier method. **a**, all stages; **b**, pathologic stage 1; **c**, pathologic stage 2; **d**, pathologic stage 3

The overall median follow-up time was 48.5±43.9 months (54.2±34.2 for LG group vs. 60.0±51.7 for OG group, *p* = 0.86). The Kaplan-Meier survival curves for the propensity score-matched cohort are shown in Figs. 3 and 4. The LG group had significantly better survival than the OG group, either OS (*p* = 0.03) or DFS (*p* = 0.01). In subgroup analysis by stages, LG continued to have better OS and DFS than OG. However, none of these differences reached statistical significance across stages (stage 1: 5-year OS, 86.0 vs. 79.5%, *p* = 0.40, 5-year DFS, 85.9 vs. 68.6%, *p* = 0.21; stage 2: 5-year OS, 86.4 vs. 70.6%, *p* = 0.20, 5-year DFS, 81.6 vs. 63.2%, *p* = 0.20; stage 3: 5-year OS, 45.0 vs. 29.0%, *p* = 0.10, 5-year DFS, 39.0 vs. 21.3%, *p* = 0.07).

The patterns of the initial recurrences are shown in Table 3. More recurrence was observed in the OG group (42 vs. 27), with locoregional being the most frequent recurrence pattern. However, the distribution differences did not reach statistical significance.

**Table 3:** Comparison of recurrence pattern in the propensity score-matched cohort

	OG (*n*=108)	LG (*n*=108)	*p* value
Initial recurrence	42	27	
Patterns			0.33
Locoregional	16 (38.1%)	6 (22.2%)	
Distant metastasis	14 (33.3%)	13 (48.1%)	
Peritoneal carcinomatosis	12 (28.6%)	8 (29.6%)	

## Discussion

In the propensity-matched cohort, we found that LG tended to have more favorable survival than OG in treating GC without compromising safety. The survival benefit was consistent for both early and advanced stages of diseases.

Several meta-analyses on non-randomized and randomized studies have compared the short-term outcome of LG versus OG for advanced GC [24–26]. Despite the longer operative time, patients undergoing LG tend to benefit from less blood loss, faster recovery, and less morbidity. In the current study, we further showed the performance of LG in daily practice in the context of treating mostly advanced stage disease and the elderly. In the propensity score-matched cohort, 62% of patients treated by laparoscopic approach were stage 2 or above, and 51.9% were over 70 years of age (Table 1). The rate of the advanced disease and the elderly in the current study were very close to the registry data in Taiwan (68.1% and 48.9%, respectively) [20]. At this point, the rates of overall and specific morbidity for the laparoscopic approach are not statistically different from the rates of the open approach (Table 2). Also, there was a trend of better survival in the LG group. Such findings may justify the routine application of LG in daily practice. The practice of applying LG for advanced GC on a large scale had been reported by studies from high-volume centers in Asia, which showed favorable short- and long-term results [27, 28]. Equal or even superior oncologic outcome was possible when performed by experienced surgeons.

Oncologic integrity is the essential requirement for LG to be applied in GC, the efficacy of which is well-accepted in early distal GC [13, 22]. In advanced GC, it is more technically demanding to achieve adequate surgical margin and lymph node dissection. We need evidence to show that it is feasible and meets the standard of oncologic surgery, as we saw in rectal cancer surgery [29, 30]. The recent multicenter randomized controlled trials from Korea (COACT 1001) and China (CLASS-01) investigating advanced GC have demonstrated comparable 3-year DFS for LG [17, 18]. Noteworthy, in the COACT 1001 study, the noncompliance rate of D2 lymph node dissection was significantly lower in the laparoscopic arm for clinical stage III patients. Such a finding suggests that the efficacy of radical lymphadenectomy in LG could be more limited when applied to extensive lymph node metastasis, such as suprapancreatic stations. In our practice, the surgeon evaluated resectability from the beginning utilizing preoperative CT scans and diagnostic laparoscopy. The procedure would be converted to open if radical lymphadenectomy was technically difficult by laparoscopic approach since conversion to open surgery is unlikely to result in inferior long-term outcomes [31, 32]. In the current study, the subgroup analysis for pathologic stage III disease showed that the 5-year DFS was even better for the LG group (39.0 vs. 18.8%, *p* = 0.06). In addition, the LG group had fewer overall recurrences than the OG group, with the dominant pattern of recurrence being distant metastasis rather than peritoneal carcinomatosis. Such results may help alleviate the concern of peritoneal seeding by pneumoperitoneum. Overall, LG was oncologically safe when resectability was properly evaluated.

The reduced surgical trauma by minimally invasive approach may not only result in faster postoperative recovery but better outcomes overall. One recent randomized controlled trial on patients who underwent neoadjuvant chemotherapy for advanced GC showed that patients in the LG group were more likely to complete adjuvant chemotherapy and less likely to terminate because of adverse effects [33]. Though the 3-year survival data were pending, one could postulate a better chance of cure with completed courses of adjuvant chemotherapy. In our experience, the better survival in the LG group in the current study could also be attributed to better tolerance of adjuvant therapy. Patients who underwent LG tended to have a faster recovery and better performance postoperatively than those who underwent OG. The better oncologic outcome achieved by laparoscopic surgery had also been observed in colon cancer [34, 35]. The level of serum interleukin-6, which has been shown to be an independent prognostic biomarker for survival in colon cancer, was lower after laparoscopic surgery [36, 37]. The reduced requirement of blood transfusion by laparoscopic approach may explain the better oncologic outcome as well [38].

We adopted LG as the primary surgical procedure for advanced GC as experience accumulated during the study period (Fig. 1). LG for GC has to meet the basic principles, including adequate surgical margin and complete lymphadenectomy according to the tumor’s location and extent [22]. For tumors at the proximal stomach, most early cases in our cohort were done by the open approach since total gastrectomy was technically demanding. Moreover, for bulky tumors with possible adjacent organs’ involvement, the surgeon decided on an open approach during the initial laparoscopic staging in the early cases. As LG successfully treated more patients with advanced GC, fewer patients were designated to OG during the step of laparoscopic staging in the later period, given the faster recovery of LG. Interestingly, the operative time was decreasing over time for both OG and LG in our cohort. It could be related to more energy and auto suture devices applied in the later OG procedure as the surgeon gained experiences from the LG. Biases from the learning curve are possible but should be minimal since the learning curves’ slope was similar (Fig. 2).

Limitations of the current study come from its single-center, retrospective design. The number for comparison in the study cohort was relatively small. The propensity score matching analysis might not fully consider residual biases associated with patient selection. For example, tumors with direct invasion outside of the stomach or bulky lymph nodes metastasis might have been excluded from the laparoscopic approach in the first place. However, they still existed in the open approach cohort, which makes allocation bias. Also, the regimen of adjuvant chemotherapy and the course of treatment were not standardized. However, the rate of advanced disease and the frequency of elderly patients within this cohort were relatively close to the general population’s true incidence. We believe this may provide surgeons with the outcome of LG in real-world practice.

In conclusion, LG can be applied to most resectable GCs, and either an advanced stage of disease or old age should not be contraindications. Patients who undergo LG could not only benefit from faster postoperative recovery but also more favorable oncologic outcomes.

## Data Availability

The data that support the findings of this study are available on request from the corresponding author, DC Chan. The data are not publicly available due to restrictions in the use of protected health information.

## Abbreviations

ASA: American Society of Anesthesiologists
BMI: Body mass index
CT: Computed tomography
DFS: Disease-free survival
ECOG: Eastern Cooperative Oncology Group
GC: Gastric cancer
LG: Laparoscopic gastrectomy
OG: Open gastrectomy
OS: Overall survival
